# Structural diversity of biologically interesting datasets: a scaffold analysis approach

**DOI:** 10.1186/1758-2946-3-30

**Published:** 2011-08-08

**Authors:** Varun Khanna, Shoba Ranganathan

**Affiliations:** 1 Department of Chemistry and Biomolecular Sciences and ARC Centre of Excellence in Bioinformatics, Macquarie University, Sydney, Australia; 2 Department of Biochemistry, Yong Loo Lin School of Medicine, National University of Singapore, Singapore

## Abstract

**Background:**

The recent public availability of the human metabolome and natural product datasets has revitalized "metabolite-likeness" and "natural product-likeness" as a drug design concept to design lead libraries targeting specific pathways. Many reports have analyzed the physicochemical property space of biologically important datasets, with only a few comprehensively characterizing the scaffold diversity in public datasets of biological interest. With large collections of high quality public data currently available, we carried out a comparative analysis of current day leads with other biologically relevant datasets.

**Results:**

In this study, we note a two-fold enrichment of metabolite scaffolds in drug dataset (42%) as compared to currently used lead libraries (23%). We also note that only a small percentage (5%) of natural product scaffolds space is shared by the lead dataset. We have identified specific scaffolds that are present in metabolites and natural products, with close counterparts in the drugs, but are missing in the lead dataset. To determine the distribution of compounds in physicochemical property space we analyzed the *molecular polar surface area*, the *molecular solubility*, the *number of rings *and the *number of rotatable bonds *in addition to four well-known Lipinski properties. Here, we note that, with only few exceptions, most of the drugs follow Lipinski's rule. The average values of the *molecular polar surface area *and the *molecular solubility *in metabolites is the highest while the *number of rings *is the lowest. In addition, we note that natural products contain the maximum *number of rings *and the *rotatable bonds *than any other dataset under consideration.

**Conclusions:**

Currently used lead libraries make little use of the metabolites and natural products scaffold space. We believe that metabolites and natural products are recognized by at least one protein in the biosphere therefore, sampling the fragment and scaffold space of these compounds, along with the knowledge of distribution in physicochemical property space, can result in better lead libraries. Hence, we recommend the greater use of metabolites and natural products while designing lead libraries. Nevertheless, metabolites have a limited distribution in chemical space that limits the usage of metabolites in library design.

## Background

An established idea of similarity-based virtual screening is that similar structures tend to have similar properties [1]. Diversifying the compound library collection for *in silico *and *in vitro *high-throughput screening without compromising biological activity remains an active research area. Chemical space is enormous but mostly biologically insignificant [2] and therefore, uninteresting from a drug design perspective. Given the large number of currently available chemical compounds in one of the largest public databases, PubChem [3], it is impossible and irrational to screen all known compounds for potential ligands. One key methodology, fragment-based virtual screening (FBVS) or fragment-based drug discovery (FBDD), is an emerging area to identify novel, small molecules for preclinical studies. In FBDD, the starting points are small low molecular weight, drug-like fragments. Examples of such fragments are ring systems, functional groups, side chains, linkers and fingerprints.

Over the past decade, substructures contributing to drug-like or lead-like properties have governed library design [4]. In one of the pioneering works to understand the distribution of common fragments in drugs, Bemis and Murcko [5] fragmented a drug dataset (taken from the Comprehensive Medicinal Chemistry database) into rings, linkers, frameworks and side chains. Using two-dimensional topological graph-based molecular descriptors, they found 2506 different frameworks for a set of 5120 drug compounds, with the top 32 accounting for the topologies of 50% of the database compounds. They concluded a skewed distribution of molecular frameworks in drugs. Metabolite-likeness is increasingly being used as filter to design lead libraries similar to metabolites with better absorption, distribution, metabolism, elimination and toxicology (ADMET) properties [6]. Many recent studies have compared chemical space occupied by compounds of pharmaceutical interest [7-12]. Grabowski and Schneider [7] studied the molecular properties and chemotype diversity of drugs, pure natural products (NPs), and natural product derived compounds. Following the approach described by Bemis and Murcko [5], they virtually dissected the molecules into frameworks, corresponding to scaffolds and side-chains. The drug dataset was ranked most structurally diverse, followed by marine and plant derived NPs, respectively. However, in contrast to the observation of Bemis and Murcko, that only 32 frameworks form the basis of nearly 50% of the compounds in CMC drug database, they found that 160 graph-based frameworks are needed to explain the chemotype of 50% of the compounds in the Collection of Bioactive Reference Analogues (COBRA) dataset [13] which contains drug-like reference molecules for ligand-based library design. In the same year, Siegel and Vieth [8] examined a set of 1386 marketed drugs and found that 15% of the drugs are embedded within other larger drugs, differing by one or more chemical fragments while 30% of drugs contain other drugs as building blocks. Recently, Franco *et al*. [9] analyzed scaffold diversity of 16 datasets of active compounds, targeting five protein classes, using an entropy-based information metric. They found that compounds targeted to the vascular endothelial growth factor receptor kinase, followed by compounds targeted to HIV reverse transcriptase and phosphodiesterase V, are maximally diverse. On the other hand, molecules in the glucocorticoid receptor, neuraminidase and glycogen phosphorylase β datasets are least diverse. Singh *et al*. [10] employed multiple criteria to compare libraries of drugs, small molecules and NPs, in terms of physicochemical properties, molecular scaffolds and fingerprints. The degree of overlap between libraries was assessed using the R-NN curve technique and the biologically relevant chemical space occupied by various compound datasets delineated. Hert *et al*. [11] compared a comprehensive dataset of 26 million compounds (i.e. a representative sample of the full chemical space) with 25810 purchasable screening compounds, metabolites, and natural product dataset. They found that almost 1300 ring systems present in NPs are missing in current day screening or lead libraries and suggest introducing bias in screening libraries towards molecules that are likely to bind protein targets. Khanna and Ranganathan [12] compared current day drugs with toxics and metabolites and found that drugs are more similar to toxics than to metabolites in physicochemical property space distribution.

As discussed above, there are many studies analyzing the scaffolds and physicochemical properties of the various chemical datasets. However, none of the studies contains a comprehensive comparison of the compounds obtained from publically available datasets of human metabolites, toxics, drugs, natural products and currently used lead libraries. In addition, we believe that inclusion of the experimental compounds from National Cancer Institute open database and the recently released ChEMBL database would enhance our analysis and prove useful in recognizing fragments in biologically interesting compounds.

In this study, we aim to answer questions such as 1) What is the physicochemical property space distribution of compounds for the datasets under comparison? 2) Are there any pharmaceutically relevant scaffolds or fragments present in metabolites and natural products that are missing in current lead libraries? 3) Are there any preferred or frequently occurring fragments and scaffolds in these datasets? 4) What is the percentage similarity of the scaffolds and fragments found in drugs to those found in other datasets?

We found patterns of commonly occurring fragments using extended connectivity functional class fingerprint (FCFP_4; details in Methods section). FCFP is a variant of extended connectivity atom type (ECFP) fingerprint, differing from the latter in the assignment of initial code [14]. The highly specific initial atoms types in ECFP fingerprints are replaced with more general atom types, with functional meaning in the FCFP fingerprints. For example, a single initial code is assigned for all halogen atoms in the FCFP fingerprints as they can often substitute each other functionally. In accord with their definition, ECFP fingerprints are a better choice to measure diversity. Therefore, we used ECFP fingerprints for diversity analysis while the more generic FCFP fingerprints were selected for Tanimoto analyses.

## Results and discussion

Five different types of pharmaceutically relevant public molecular datasets were selected for this study: drugs, human metabolites, toxics, natural products and a sample of currently used lead compounds. Furthermore, we have also considered two popular small molecule databases *viz*. National Cancer Institute (NCI) database and ChEMBL database (details in the Methods section). Our results are presented in three sections, *viz*. preliminary analysis (measuring diversity and Tanimoto similarity), calculating physicochemical properties and scaffold analysis.

After carefully pruning and filtering the datasets, all the datasets were clustered (see Methods section) to avoid biased results due to overrepresentation of similar molecules.

### 1. Preliminary analysis

#### 1.1 Diversity analysis

**Figure 1:**
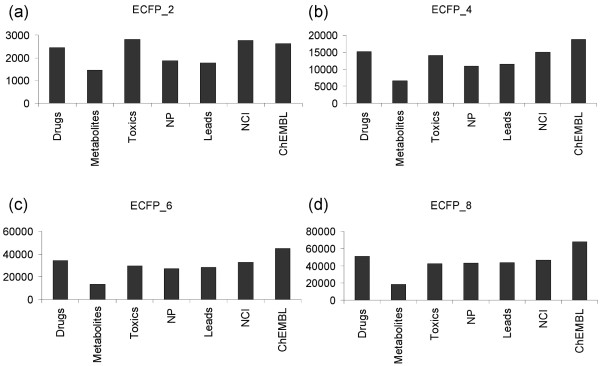
**The number of non-redundant fingerprint features as a function of ECFP fingerprint order**. Fingerprints of orders 2, 4, 6 and 8 for datasets comprising drugs, metabolites, toxics, natural products, leads, NCI and ChEMBL are presented.

In order to compare the diversity of features (fragments) present in each dataset, we have plotted the total number of non-redundant fingerprint features calculated, using ECFP fingerprints, up to order 8 (Figure 1). Our results indicate that overall, the ChEMBL dataset generates the maximum number of fragments and is highly diverse, while the metabolite dataset is the least diverse. From Figure 1a, we note that initially toxics outnumber other molecular datasets in generating features. This could be due to the high heteroatom content in toxics, resulting in large numbers of ECFP features generated during the first iteration step of fingerprinting. Similarly, the NCI dataset contains a large number of features during the initial iteration step of fingerprint feature generation. Metabolites, on the other hand, produce the least number of features, which suggests a limited occupancy of chemical space. Drugs were moderately diverse throughout and we find an increase in fragment diversity with increasing order of fingerprints.

#### 1.2 Tanimoto analysis

The Tanimoto similarity coefficient compares two molecules, A and B, having *N_A _*as the number of features in A, *N_B _*as the number of features in B, and *N_AB _*as the number of features common to both A and B as given in equation 1. This value is usually reported in the binary form, represented as *T_b_*, and reported for simple comparisons between molecules. However, the Tanimoto coefficient can also encompass nonbinary data [15]; for example, if a fingerprint encodes not just the fragment incidences but also the frequencies of occurrence, as in the case of comparison between two compound datasets. In this case, the Tanimoto coefficient (*T_nb_*) is given by equation 2 where *x_i_A, x_i_B *are the number of times the *i*th fragment occurs in A and B, respectively, summed over *n *elements of each fingerprint.(1)(2)

We extend this concept to compare different datasets used in this study. To calculate how similar two datasets are, we first calculated the Scitegic Pipeline Pilot connectivity fingerprints, FCFP_4 (details in the Methods section) for all the datasets. Subsequently, the sum of squares of the frequency of fingerprint features was calculated over the *n *elements for each dataset. Finally, the common features present in both datasets were counted and their frequencies multiplied, to determine *T_nb_*.

For the five different datasets described in the Methods section, as well as the two reference datasets, NCI and ChEMBL, the Tanimoto coefficient values are shown in Table 1. We note that the FCFP fingerprint patterns (of order 4; FCFP_4) found in drugs are most similar to toxics (FCFP_4: 0.91) than to any other dataset, except for the fingerprint patterns found in reference datasets. On the other hand, drugs are least similar to metabolites (FCFP_4: 0.72). These observations are consistent with our earlier study on smaller datasets [12]. We also note that ChEMBL contains more drug-like fragments than any other biologically relevant fragment type present in this study (FCFP_4: 0.94). Further, we note that the fragments found in metabolites are least similar to the fragments present in NPs and lead dataset. Additionally, with the increasing order of fingerprints (FCFP_6 and so on), although the number of fragments generated increases, the Tanimoto similarity coefficient values fall slightly for all the datasets compared (data not shown). This suggests an inverse relationship between the size of the fragment and the probability of its occurrence in two separate datasets, i.e. the larger the fragment, the less likely that it will be found in the two datasets being compared.

**Table 1:** Tanimoto similarity values using circular connectivity fingerprint descriptors for different datasets under study.

Datasets	Drugs	Metabolites	Toxics	NPs	Leads	NCI	ChEMBL
**Drugs**	1	0.72	0.91	0.85	0.78	0.92	0.94
**Metabolites**		1	0.73	0.58	0.49	0.67	0.63
**Toxics**			1	0.80	0.75	0.94	0.84
**NPs**				1	0.76	0.84	0.88
**Leads**					1	0.85	0.88
**NCI**						1	0.90
**ChEMBL**							1

The upper half of the diagonal contains non-binary Tanimoto similarity values calculated using the FCFP_4 fingerprint.

### 2. Physicochemical property analysis

#### 2.1 Lipinski's properties for "rule of five" (Ro5) compliance

Ro5 has dominated drug design since 1997 and therefore, we believe it would be useful to analyze these datasets for compliance with the Ro5 test. Ro5 predicts passive and oral absorption based on log P, molecular weight, hydrogen bond donors and hydrogen bond acceptors. We report in Table 2, the percentage of molecules "failing" the Ro5 test, i.e. at least not meeting one condition of the Ro5 test. The results are comparable for both kinds of datasets, showing that randomly selected subsets are representative of the clustered datasets. Also, for the clustered datasets, initially, over 25% of drugs do not adhere to Ro5 while 68% of the metabolites are outside Lipinski's universe. However, by removing lipids from metabolites we note that the percentage of molecules failing Ro5 test drops to 20% indicating that majority of the lipids do not follow Lipinski's rule. Further, we found that similar to drugs, only 26.5% of the toxics fail the Ro5 test. Lipinski's rule was originally designed to estimate bioavailability of compounds rather than toxicity. Therefore, the above result suggests that empirical rules such as Ro5 can be supplemented with toxicity information in order to reduce high attrition rates during drug discovery programs as has been reported in the literature [16,17]. Further, we found that only 16% of NPs failed Lipinski's test. Many other related studies on NPs have reported similar results [7,18]. Grabowski and Schneider [7] analyzed pure natural products (isolated exclusively from plants and terrestrial microorganisms) from MEGAbolite and Interbioscreen, natural products derivatives (isolated and synthesized natural products and derivatives from natural sources like plants, fungi, bacteria and sea organisms) from BioSpecs and marine natural products (isolated from sponges (41%), Coelenterates (21%), marine microorganisms and phytoplankton (10%)) from the literature. They found that 18% of the pure natural products, 30% of the marine natural products and only 8% of the natural product derived compounds violate Lipinski's rule, averaging 18.7%. While Grabowski and Schneider have reported results very similar to ours, Ganesan [18] analyzed a focused set of 24 natural products that were the starting point for marketed drugs in the 25-year period from 1981-2006 and found that 50% of these failed Lipinski's rule. In general, NPs do not necessarily abide by Lipinski's rule because they are thought to enter the human body not by passive diffusion but by more complex mechanisms such as active transportation, and so are not expected to comply with the rules for bioavailability. The probable explanation of our results could be the manner in which the NP dataset is pooled at the ZINC database. ZINC is a public database for commercially available compounds and NPs present in ZINC are pre-filtered to cover more drug-like space, contributing towards Ro5-like characteristics. Lead molecules on the other hand also did well in the Ro5 test as only 19.5% of the molecules violated one or more than one condition of the Lipinski's rule. This is in accordance with the lead-likeness concept proposed earlier [19] which states that leads should be simple, low molecular weight molecules and thus, should fall well within Lipinski's universe. Further, our results indicate that, NCI compounds follow Lipinski's rule more strictly than compounds present in ChEMBL dataset.

**Table 2:** Comparision of the number of molecules failing Lipinski's "rule of five" (Ro5) in clustered and randomly selected datasets.

Dataset	Total no. of molecules (in clustered dataset)	% of molecules failing Ro5 in clustered datasets	% of molecules failing Ro5 in randomly selected subset
**Drugs**	3788	25.7	23.0
**Metabolites**	6124	68.0	20.0*
**Toxics**	2166	26.5	21.5
**NPs**	61972	16.2	15.0
**Leads**	67983	19.8	19.5
**NCI**	161336	19.5	15.5
**ChEMBL**	379827	36.4	36.0

*Metabolite dataset excluding lipids and large molecules (details in the Methods section)

#### 2.2 Lipinski's properties as boxplots

**Figure 2:**
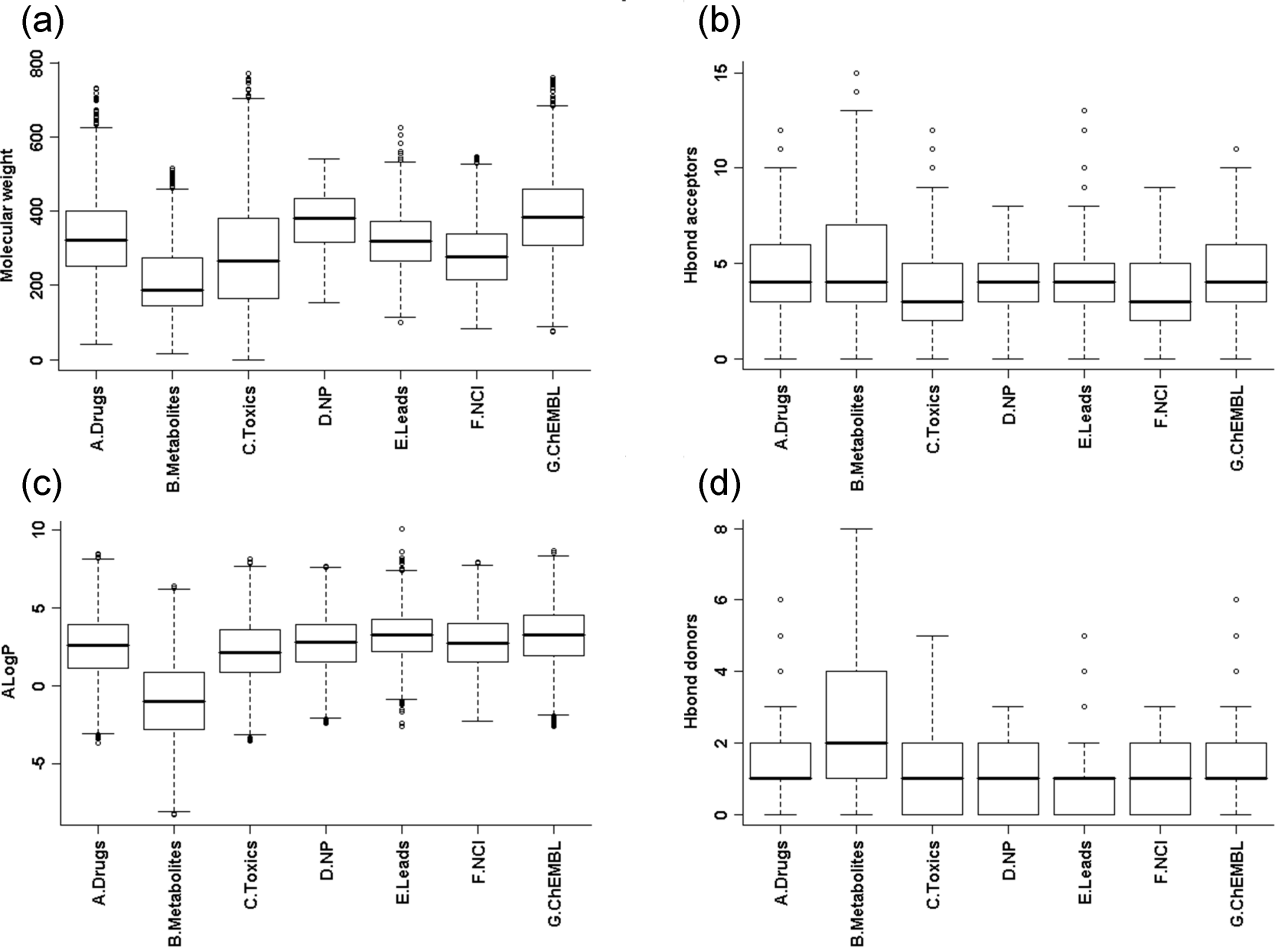
**Box plots for the Lipinski physicochemical properties**: (a) molecular weight, (b) the number of hydrogen bond acceptors, (c) AlogP and (d) the number of hydrogen bond donors.

Box plots for Lipinski properties for random subsets are available from Figure 2. We find that the mean value for the molecular weight in the metabolite dataset is relatively low when compared to the other datasets such as drugs, leads and natural products. We also observe that the lead dataset is well within Lipinski's universe and covers a fair amount of drug space. Further, we find a noticeable difference in lipophilicity values of metabolites as compared to drugs and leads. The mean value of lipophilicity (measured as AlogP) suggests that metabolites prefer a hydrophilic environment. Our results are comparable to the recent study using similar datasets [6]. In this study, lipophilicity (measured by a similar parameter, clogD) for drugs, metabolites and library compounds showed that the distribution of library compounds is similar to that of drugs, but differ markedly from metabolites and that metabolites are more hydrophilic than both drugs and library compounds.

#### 2.3 Other physicochemical properties

**Figure 3:**
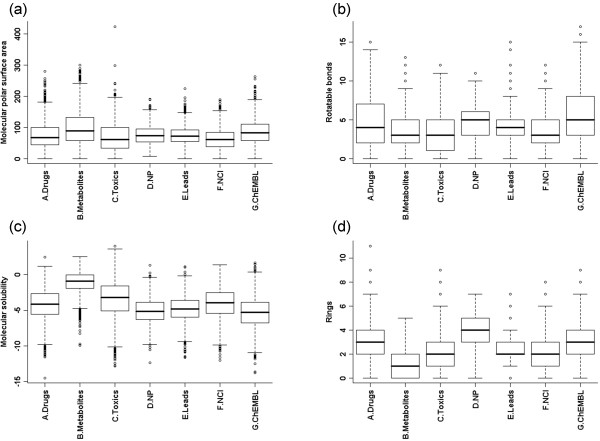
**Box plots for other significant physicochemical properties**: (a) molecular polar surface area, (b) the number of rotatable bonds, (c) molecular solubility and (d) the number of rings.

For a comprehensive study on the physicochemical property space distribution, we computed four more common whole molecule descriptors: the *molecular polar surface area*, the *number of rotatable bonds*, the *molecular solubility *and the *number of rings *(details in the Methods section). Distributions of these physicochemical properties as box plots are available from Figure 3. We note that metabolites show relatively higher solubility, higher molecular polar surface area but lower complexity (less rings, less rotatable bonds and lower molecular weight) compared to drugs. Further, our results indicate that, in general, NCI molecules are also low molecular weight compounds with less complexity and slightly higher solubility than drug molecules. In addition, we note that a large part of the ChEMBL database contain drug-like compounds with a biasness towards higher molecular weight and more complex molecules than drugs.

### 3. Scaffold or cyclic system analysis

It is quite informative to study the molecular frameworks while comparing different datasets of chemical compounds. Since the publication of Bemis and Murcko [5], many attempts have been made to explore the chemical space occupied by bioactive scaffolds [20] as scaffold hopping remains an active area under research [21]. In this study, we define scaffolds as the core structure of the molecule after removing side chains but not the linkers, similar to the definition of *atomic frameworks *used by Bemis and Murcko. A detailed analysis of the total number of non-redundant scaffolds present in the different datasets is available in Table 3. The percentage of singletons (scaffolds occurring only once) relative to the total number of scaffolds in a dataset has also been reported. In addition, we have tabulated the proportion of non-redundant scaffolds containing aromatic and non-aromatic rings.

**Table 3:** Scaffold analysis of various clustered datasets under study.

Dataset	Occurrence of scaffolds (% relative to dataset size)		No. of singletons (% relative to number of scaffolds)		Aromatic scaffolds (% relative to number of scaffolds)	
	
	**No**.	%	**No**.	%	**No**.	%
**Drugs**	1874	50.0	1411	75.3	1588	85.0
**Metabolites**	296	14.3	181	61.1	140	47.3
**Toxics**	905	42.0	689	76.1	656	72.3
**NPs**	13151	21.2	6053	46.0	11776	90.0
**Leads**	21621	32.0	13819	64.0	21057	97.4
**NCI**	44324	28.0	31880	72.0	36778	83.0
**ChEMBL**	126843	33.4	87750	69.2	119419	94.1

Frequency of occurrence for non-redundant scaffolds (relative to the dataset size) and number of aromatic ring containing scaffolds (relative to the total number of non-redundant scaffolds) have been reported.

The drug dataset generates the largest proportion of non-redundant scaffolds (50.0%) relative to the dataset size, followed by the toxics (42%), ChEMBL (33.4%), leads (32%) and NCI dataset (28%). Exceptionally low number of scaffolds in metabolites (14.3%) and natural products (21.2%) suggest lower scaffold diversity in these datasets. The higher scaffold diversity in drugs could be attributed to the fact that drugs are derived from various biologically relevant compounds. The drug scaffold diversity is probably also due to the patenting requirements, to position functionality in the same way as an existing drug but outside of its patent space, that is often achieved by a minor change in the scaffold. Similarly, a large number of scaffolds in the toxic compound set is indicative of the high diversity of compounds with toxicity potential. Further, we note that distribution of scaffolds in all the datasets in highly skewed with large number of them occurring only once (singletons). In fact, almost 70% of the scaffolds in drugs, toxics, NCI and ChEMBL dataset occur only once. We also found that natural products comprise maximum number of recurring scaffolds (100 - % of singletons = 64%) followed by metabolites (38.9%) and leads (35.7%) suggesting that the compounds in these datasets revolve around certain preferred types of scaffolds. Our results agree with the recent study using similar natural product and drug dataset [10]. In their study, authors found high scaffold diversity in drugs (39.7%) while low diversity in natural products (17.9%) which is in accordance with our results. By counting the number of aromatic rings in non-redundant scaffolds, we note that metabolites contain least number of aromatic rings (only 47.3% contain one or more aromatic rings in a scaffold) as compared to other datasets. 85% of the drugs on the other hand have scaffolds with aromatic rings. Furthermore, we note that 97.4% of the scaffolds found in lead dataset contain aromatic rings. There seems to be a bias towards aromatic ring containing scaffolds in presently used lead libraries.

**Figure 4:**
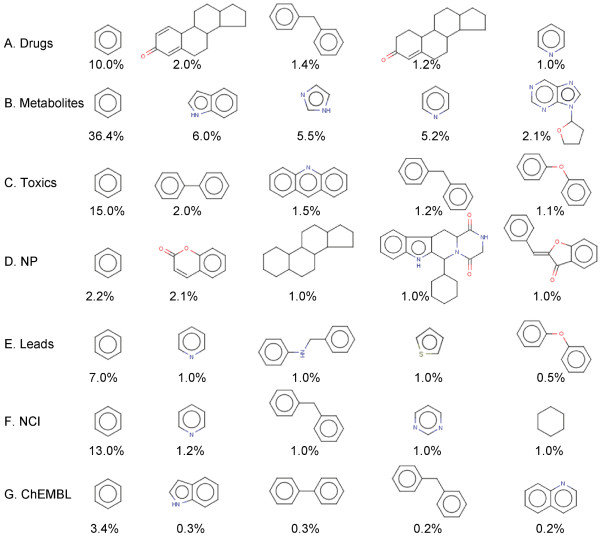
**Top 5 scaffolds derived from A. drugs, B. metabolites, C. toxins, D. natural products, E. leads, F. NCI and G. ChEMBL**. The extent of occurrence of the scaffold relative to the total number of scaffolds in the dataset (as %) are listed.

The top five scaffolds and their relative percentages based on the total number of scaffolds found in each dataset are shown in Figure 4. Benzene is the most abundant scaffold system in all the datasets, particularly in metabolites (over 36%). Apart from metabolites, toxics (15%) and NCI compounds (13%) also contain benzene in high percentages. Drugs and leads, on the other hand contain benzene in moderate amounts (10% and 7% respectively). While benzene is the most common scaffold type in NP (2.2%) and ChEMBL datasets (3.4%), the relative abundance of benzene in these datasets is far lower than that in the other datasets. Following benzene, pyridine is the second most commonly occurring scaffold type in the top five scaffolds. It is found in four out of seven datasets: metabolites (5.2%), drugs (1%), leads (1%), and NCI (1.2%). We also note that steroid derivatives are largely present in drugs and NPs. Similarly, most of the fused large scaffolds are found in NPs (four of the top five scaffolds) followed by drugs and the ChEMBL dataset. Metabolites, on the other hand, seem to prefer smaller, less complex systems. Likewise, toxics and lead compounds also have few complex fused systems. Other commonly occurring scaffold systems are purine and purine derivatives (found mainly in metabolites and ChEMBL dataset), imidazole and biphenyls.

In Table 4, we tabulate the percentages of non-redundant shared scaffolds between pairs of different datasets. From Table 4 we note that drugs and metabolites share 6% of the total non-redundant scaffolds whereas NPs, leads and toxics share overall 2.4%, 1.4% and 7.5% of scaffolds with drugs, respectively. It is interesting to note that metabolites and leads do not share as many scaffolds (0.3%) as drugs and metabolites (6%). Due to the uneven size of the datasets, we have also reported the contribution of each dataset to the set of shared scaffolds. We find that of the total 296 non-redundant scaffolds found in metabolites (Table 3), 123 (42%) are shared by drugs whereas only 68 (23%) are shared by the lead dataset. This suggests that lead compounds need further optimization to become more metabolite-like. Similarly, there seems to be little overlap between the scaffolds of presently used lead libraries and NPs (2.1%). Since metabolites and NPs are recognized by at least one protein in the biosphere, they seem to be appropriate candidates in lead library design. Our results however, indicate that neither metabolites nor NP scaffolds are being sampled enough while designing lead libraries. In addition, we note that over 7% of scaffolds are shared between drugs and toxics while metabolites and toxics share over 6% of the scaffolds, suggesting the recurrence of common scaffolds between these datasets. Compounds in the NCI and ChEMBL datasets are quite diversified; however, the NCI dataset clearly contains more toxic scaffolds than the ChEMBL dataset. Furthermore, we note that large part of the drug scaffold space is present in NCI (45%) and ChEMBL (72%) implying that these datasets cover good amount of drug-like compounds. We also note that a large part of metabolite scaffold space is present in natural product (47%), NCI (78%) and ChEMBL (73%) datasets.

**Table 4:** Scaffolds shared between pairs of clustered datasets.

Datasets	D	M	T	P	L	N	C
**D**	100%	123 (6%; D: 7%, M: 42%)	192 (7.5%; D: 10%, T: 21%)	347 (2.4%; D: 19%, P: 3%)	310 (1.4%; D: 17%, L: 1%)	840 (2%; D: 45%, N: 2%)	1347 (1.0%; D: 72%, C: 1%)
**M**		100%	71 (6.3%; M: 24%, T: 8%)	140 (1.1%; M: 47%, P: 1%)	68 (0.3%; M: 23%, L: 0.3%)	230 (0.5%; M: 78%, N: 0.5%)	215 (0.2%, M: 73%, C: 0.2%)
**T**			100%	174 (1.3%; T: 19%, P: 1%)	144 (0.7%; T: 16%, L: 1%)	534 (1.2%, T: 59%, N: 1%)	532 (0.4%, T: 59%, C: 0.4%)
**P**				100%	706 (2.1%; P: 5%, L: 3%)	1734 (3.1%; P: 13%, L: 8%)	1947 (1.4%, P: 15%, C: 1.5%)
**L**					100%	2753 (4.4%; L: 13%, N: 6%)	3470 (2.4%; L: 16%, C: 3%)
**N**						100%	7600 (5.0%; N: 17%, C: 6%)
**C**							100%

The overall percentage of shared scaffolds is given in the brackets, along with percentages of shared scaffolds from each contributing dataset.

D: Drugs, M: Metabolites, T: Toxics, P: Natural Products, L: Leads, N: NCI, C: ChEMBL.

**Figure 5:**
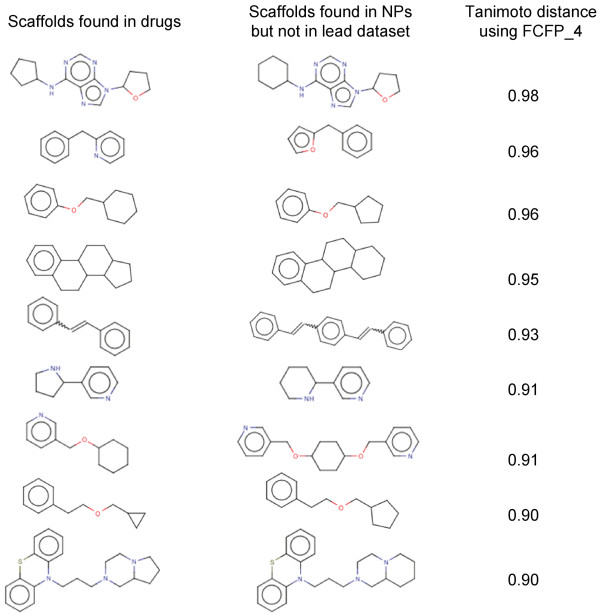
**A set of scaffolds present in NPs but are missing in lead dataset**. The Tanimoto distance of these scaffolds with the closest counterparts in drugs is also reported.

**Figure 6:**
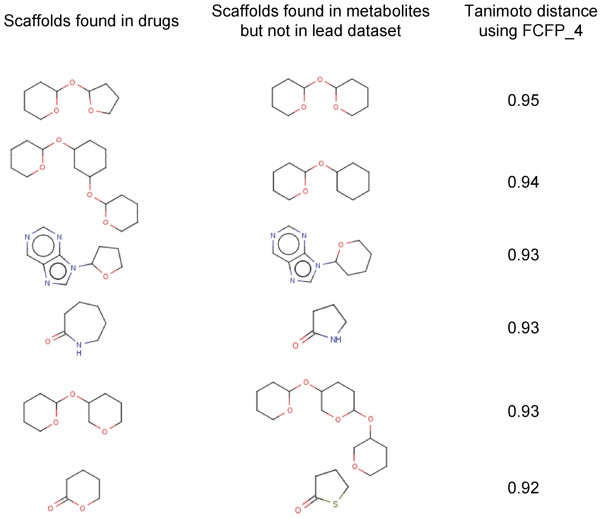
**A set of scaffolds present in metabolites but are missing in lead dataset**. The Tanimoto distance of these scaffolds with the closest counterparts in drugs is also reported.

We expect that lead libraries biased towards molecules that biological targets have evolved to recognize, would yield better hits rates, than unbiased or universal libraries. Metabolites and NPs could potentially provide suitable lead molecules. Consequently, we further analyzed these datasets for the type of scaffolds that are currently missing in lead libraries. In fact, we note a very slight overlap in the scaffold space of lead libraries and these datasets as discussed above. We therefore, suggest that with the optimum coverage of biologically relevant scaffold space, hit rates in high throughput screening experiments can be improved. We report a set of scaffolds that occur in NPs (Figure 5) and metabolites (Figure 6), with a minimum Tanimoto similarity of 0.9 to the scaffolds found in drugs, which are actually missing in currently used lead datasets.

## Conclusions

In this study, we have carried out a detailed analysis of commonly occurring fragments in various datasets of biological interest. Dataset comparison using the Tanimoto coefficient shows that drugs and toxics share a large number of topological fragments whereas drugs are least similar to metabolites than to any other dataset studied. However, in scaffold analysis we found that drugs and metabolites share 6% of the total non-redundant scaffolds, i.e. over 42% of the metabolite scaffolds are present in drugs, whereas only 23% of the metabolite scaffolds are represented in current leads. This shows that although drugs and metabolites share many scaffolds, they largely differ in topological fragment space. Further, we conclude that current lead libraries do not cover much of metabolite scaffold space.

Library design is a multi-class optimization problem. It often presents a trade-off between several factors, including diversity and ADMET properties. Since metabolites and NPs are already optimized by millions of years of evolution to bind to at least one biological macromolecule therefore, it is highly likely that libraries designed based on the scaffolds and fragments occurring in metabolite and NP space will result in molecules with better ADMET properties. Hence, the use of metabolites and NPs while designing lead libraries would be beneficial. However, metabolites occupy a limited space in chemical universe that limits their usage in library design.

From physicochemical properties analysis, we note that there is a need to diversify present day lead libraries in order to optimize the coverage of chemical space. We also note that with the exception of few compounds, most of the drug molecules follow Lipinski's rule whereas over 68% of metabolites are outside Lipinski's universe. On a closer examination of metabolites, we found that the compounds that do not follow Lipinski rule are mainly lipids and large molecules. Further, we note that lipid-free metabolite dataset contains low molecular weight and less complex molecules as compared to other datasets. Our studies on scaffolds systems suggest that drugs are most diverse (50% scaffolds relative to the dataset size) and prefer aromatic to non-aromatic ring-containing scaffolds. Metabolites, on the other hand, have a very narrow distribution of scaffolds (only 14.3% scaffolds relative to the dataset size) of which 38.9% recur. The exceptionally low number of cyclic systems in metabolites implies lower scaffold diversity in metabolites. Further, we confirm earlier reports of skewed distribution of scaffolds, with many more singletons than recurring scaffolds.

## Methods

### Preparation of datasets

Five different types of biologically relevant molecular datasets have been considered in this study. Beside these, the contents of public databases like NCI and ChEMBL were also analyzed. Table 5 presents a summary of all the databases used in this study. The drug dataset was assembled by merging molecules obtained from the DrugBank [22] and a subset of Kyoto Encyclopedia of Genes and Genomes database (KEGG DRUG) [23]. DrugBank is a comprehensive resource on drugs and includes over 1350 FDA-approved small drugs. KEGG is a bioinformatics resource and currently provides 19 databases; we used the KEGG DRUG subset as it contains all the drugs approved in the USA and Japan. It not only contains prescription drugs but also "over-the-counter" (OTC) drugs. Similarly, for metabolite dataset we used the Human Metabolome Database (HMDB) [24], HumanCYC [25] database and BiGG [26] database. HMDB contains information on nearly 8,000 metabolites found in the human body. HumanCYC is a bioinformatics database that combines human metabolic pathway and genome information, providing KEGG, PubChem and ChEBI identifiers for the metabolites present in this database. BiGG stores manually annotated human metabolic network information, with links to KEGG metabolites.

**Table 5:** Databases used in this study

Datasets		Number of molecules	Clustered dataset	Reference
Drugs	DrugBank	1372	3788	[22]
	KEGG drugs	7057		[23]
Metabolites	HMDB	7888	6124, 2072*	[24]
	HumanCYC	984		[25]
	BiGG	730		[26]
Toxics	DSSTox	582	2166	[27]
	FDA Carcinogenicity	125		[28]
	ITER	514		[30]
	SuperToxic	1097		[31]
NPs	ZINC NP database	89425	61972	[32]
Leads	BioNET	42699	67983	[33]
	Maybridge	60550		[34]
NCI	NCI database	260071	161336	[39]
ChEMBL	ChEMBL dataset	600625	379827	[36]

*Metabolite dataset excluding lipids and large molecules (details in the Methods section).

Likewise, for the toxics dataset, compounds from various public sources were integrated to make a single dataset focusing largely on carcinogenic molecules. The Distributed Structure-Searchable Toxicity (DSSTox) Carcinogenic Potency Database [27] contains experimental results and carcinogenicity information for 1547 substances tested against different species. Contrera *et al*. [28] published a dataset of 282 human pharmaceuticals obtained from FDA database for carcinogenicity studies on mouse and rat. They reported 125 (44% of the above 282) of the positive chemicals that were used in this study. Toxicology Excellence for Risk Assessment (TERA) is an independent non-profit organization dedicated to the public health. Since 1996, TERA has maintained an International Toxicity Estimate for Risk database [29] which provides chronic human risk assessment data from organization around the world for over 650 chemicals [30]. Finally, ~1000 molecules with medium and high toxicity were downloaded from the SuperToxic database [31]. The dataset for NPs was obtained from the ZINC database [32]. These molecules can be searched under the subset tab, as "Meta subsets". For lead dataset, we merged two independent screening sets obtained from BioNET [33] and Maybridge database [34]. The molecules in these two databases are well diversified and we integrated them to form a dataset of lead compounds as found in pharmaceutical collections. Further, we included molecules from NCI open database [35]. The latest September 2003 release of the database stores 260071 organic compounds tested by NCI for anticancer activity. Since many of the compounds are experimental, have not been tested for human consumption and covers high diversity therefore, we believe it would be good choice to include this dataset in our study. One other public dataset, ChEMBL [36] was used as the reference dataset for biologically interesting molecules. ChEMBL is a chemogenomics data resource with over 8000 targets and about 622,884 bioactive compounds.

All datasets are current as of 10-November-2010.

### Cleaning and processing of the datasets

We followed a standard cleaning procedure (see additional file 1) to obtain a non-redundant dataset in each category. Finally, clustering was performed to address the issue of possible overrepresentation of the chemical space, which might bias the analysis results towards similar molecules [6]. Clusters were generated, using the Cluster "Clara" algorithm embedded in the Pipeline Pilot (PP) software [37] by employing an atom type fingerprint as a chemical descriptor and Euclidean distance was the distance metric selected. Cluster centers served as the representatives for clusters containing more than one molecule while singletons were directly used as cluster centers. This resulted in 30% decreases of each dataset. Upon further analysis, we found that clustered metabolite set contains lipids in large numbers. In order to remove the bias towards lipids and large molecules, we filtered out lipids resulting in 2072 molecules in the "lipid-free" metabolite dataset, used for analysis in this study.

To simplify the analysis, we randomly selected 2000 compounds from each of the clustered datasets and lipid-free metabolite dataset in case of metabolites. The majority of the analysis was carried out using the clustered datasets and lipid-free metabolite dataset, except for preliminary analysis, where these randomly selected molecules were used and in the case of Ro5 test, where both datasets were compared.

### Molecular descriptors

All the descriptors were calculated using PP. Beside the four Lipinski properties: molecular weight, the number of hydrogen bond acceptors, AlogP (a hydrophobicity measure) and the number of hydrogen bond donors [4], other descriptors such as molecular polar surface area (MPSA), molecular solubility (MS), the number of rings (NR) and the number of rotatable bonds (NRB) were also computed. AlogP was calculated using the Ghose-Crippen method [38] which takes into account the group's contribution to Log P. MPSA is defined as the sum over all the polar atoms. This descriptor is correlated with drug transport capabilities and is important in penetrating the blood-brain barrier. The NRB is a direct measure of the flexibility of molecules thus related to MPSA. Binary descriptors (ECFP_4 and FCFP_4) were calculated using a structural property calculator embedded in PP. Initially, each atom is assigned a code based on its properties and connectivity. With increasing iteration, each atom code is combined with the code of its immediate neighbours to produce the next order code. This process is repeated until the desired number of iterations has been achieved, typically to four iterations, generating ECFP_4, or FCFP_4 fingerprints.

### Cyclic systems

In addition to examining the physicochemical properties, each dataset was also explored for the frequent scaffold systems. We used an inbuilt PP protocol to identify the most common fragments, by setting "FragmentType" to MurckoAssemblies and adjusting "MaxFragSize" parameter at the required level.

## Competing interests

The authors declare that they have no competing interests.

## Authors' contributions

VK curated the datasets and conducted the analysis work; SR directed the study and both authors prepared and approved the manuscript.

## Supplementary Material

Additional file 1 **Supplementary figure S1**. Flowchart adapted for the overall methodology. Click here for file
