# Supplementary material for: Structural diversity of biologically interesting datasets: a scaffold analysis approach
Source: J Cheminform. 2011 Aug 8;3:30. doi: 10.1186/1758-2946-3-30 (PMC3179739; doi:10.1186/1758-2946-3-30)
Supplement: Additional file 1 — Supplementary figure S1. Flowchart adapted for the overall methodology. [file 1758-2946-3-30-S1.PDF]

Additional File 1

Structural diversity of biologically interesting datasets: a scaffold analysis approach

Varun Khanna, Shoba Ranganathan

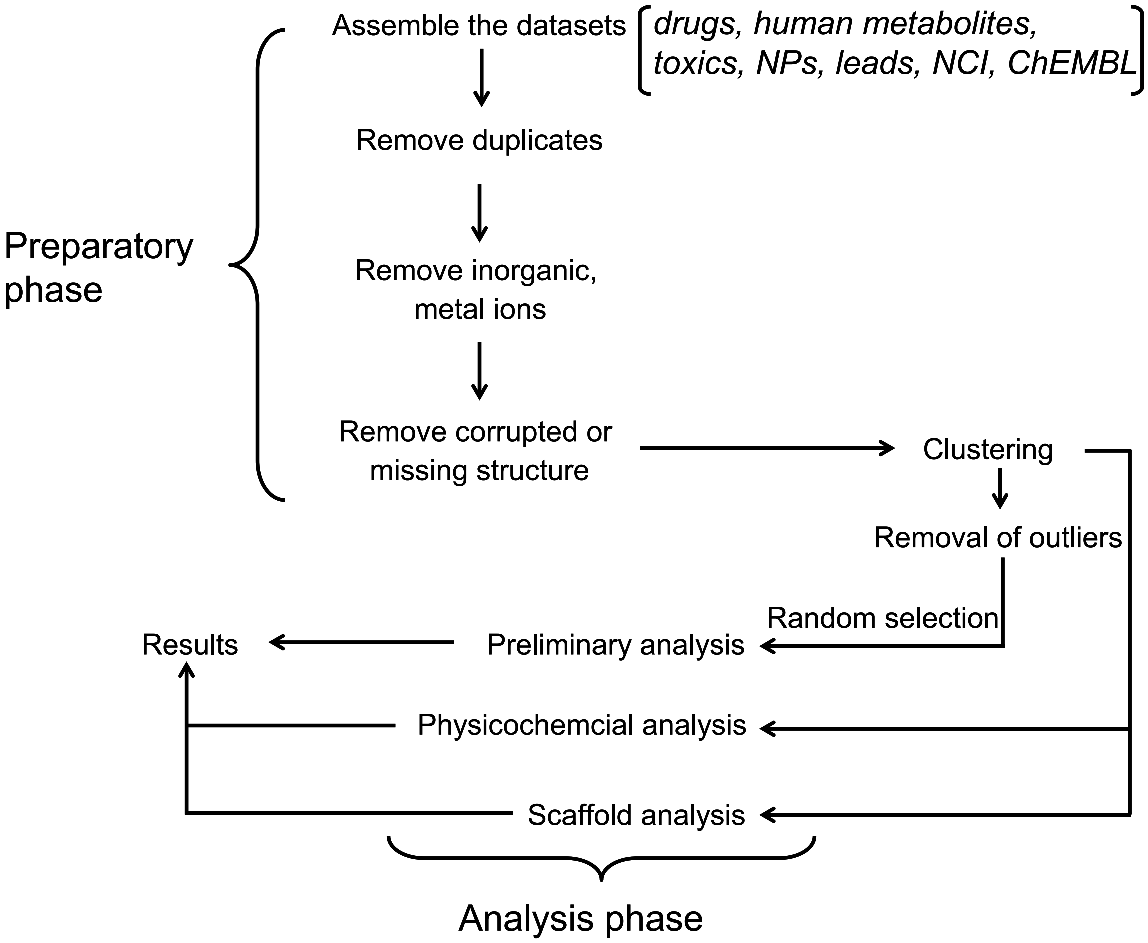

Figure S1: Flowchart adapted for the overall methodology.
